# Does self-efficacy mediate the relationship between occupational stress and mental health problems? A study among nursing professionals

**DOI:** 10.34172/hpp.2021.44

**Published:** 2021-08-18

**Authors:** Iman Dianat, Sonia Azemi, Farahnaz Abdollahazade, Ahmad Bazazan, Mohammad Asghari Jafarabadi

**Affiliations:** ^1^Department of Occupational Health, Faculty of Health, Tabriz University of Medical Sciences, Tabriz, Iran; ^2^Faculty of Nursing and Midwifery, Tabriz University of Medical Sciences, Tabriz, Iran; ^3^Center for the Development of Interdisciplinary Research in Islamic Sciences and Health Sciences, Tabriz University of Medical Sciences, Tabriz, Iran; ^4^Department of Statistics and Epidemiology, School of Medicine, Zanjan University of Medical Sciences, Zanjan, Iran

**Keywords:** Mediation analyses, Self-esteem, Mental disorders, Nurses, Psychological stresses

## Abstract

**Background:** While it is acknowledged that self-efficacy plays a significant role in understanding consequences of occupational stress, no research has given much attention to the mediating effect of self-efficacy in the association between occupational stress and mental health (MH)problems. The aims of this study were to examine: (1) the associations between occupational stress, MH problems, and self-efficacy among nursing professionals, and (2) mediating effect of self-efficacy in the association between occupational stress and MH problems.

**Methods:** A multi-hospital cross-sectional survey was adopted in eight hospitals in Tabriz, Iran. 389 nursing staff were recruited through a two-stage sampling procedure. Study variables included occupational stress (Health & Safety Executive [HSE] Management Standards RevisedIndicator Tool [MS–RIT]), mental health (General Health Questionnaire [GHQ–28]), and self efficacy (General Self-Efficacy [GSE–10]). Generalized structural equation modelling (GSEM)was applied.

**Results:** Occupational stress (mean±SD=109.2±13.4), poor MH (41.9%), and low selfefficacy (mean±SD=17.7±4.9) were fairly common among the participants. The results showed significant direct effect of occupational stress on MH problems (β=- 0.38, P<0.001). Indirect effect of occupational stress on MH problems through self-efficacy was not significant.

**Conclusion:** The findings highlight the role of other mechanisms or factors than self-efficacy in the association between occupational stress and MH problems that should be established in future work.

## Introduction

Occupational stress, as a complex concept, is a product of interaction between individuals, work environment, and cultural contexts.^1,2^ Occupational stress has been identified as one of largest problems in both developed and developing countries.^3,4^ In recent years, an increasing proportion of working population worldwide have experienced this problem.^5,6^ Occupational stress can be considered as harmful physical and psychological responses in employees resulting from lack of balance between job demands and personal abilities, need or resources.^7^ Occupational stress is associated with several negative consequences for both employees (e.g., impact on health and well-being, family and social adjustments, coping strategies, job satisfaction and performance) and organisations (e.g., intention to leave, increased staff turnover, and education/training costs to improve staff morale).^8-12^

Nurses are a group of healthcare professionals who experience a relatively high stress level.^11,12^ This is often attributable to a variety of work-related stressors including high workload, shift working, lack of adequate attention to nursing profession, low social support, conflict with physicians and bullying and violence as well as dealing with death, patients, and their families.^9,13,14^ In addition, continuous organisational changes such as development of new technologies and therapeutic methods, increasing demands and over-expectations of patients, which are essential elements of health systems, are additional sources of stress for this group of workers.^9,15,16^ The literature on stress shows that workplace stressors influence mental and psychological well-being of nursing personnel.^10,17,18^ Poor mental health (MH) conditions resulted from occupational stress in nurses could have a negative impact on their occupational performance and have an adverse effect on patient outcomes.^19^ Therefore, it is important to expand knowledge on the MH status of nursing personnel.

With regard to the multidimensional nature of occupational stress, a number of factors (including individual, work-related and organizational factors) can influence the consequences of occupational stress on employees. Self-efficacy is an individual trait which plays a significant role in understanding of consequences of occupational stress. This is particularly the case for nursing personnel, who are exposed to workplace stressors on a regular basis. Self-efficacy is defined as: “people’s judgements of their capabilities to organise and execute courses of action required to attain designated types of performances”.^20^ It can be hypothesised that the presence of various work-related stressors has a significant impact on employee’s beliefs or confidence, and consequently on their health and performance. However, review of the literature indicates that relatively few studies have examined the relationship between occupational stress and self-efficacy in nursing population. Two recent studies have reported negative association between stress and self-efficacy among nursing personnel,^21,22^ and only one of them has focused on job stress among hospital nurses.^21^ In addition, as self-efficacy plays a major role in how people react to challenging tasks (e.g., such as stressful nursing tasks), there is a need to improve the knowledge on how this individual trait (e.g., self-efficacy) influences the effect of occupational stress on MH status of nursing staff. Research to be conducted on this issue will help to better understand the role of individual characteristics on the relationship between occupational stress and MH profile of employees. Nevertheless, a review of the literature indicates that no research has given much attention to the mediating effect of self-efficacy in the association between occupational stress and MH.

### Conceptual framework

**Figure 1:**
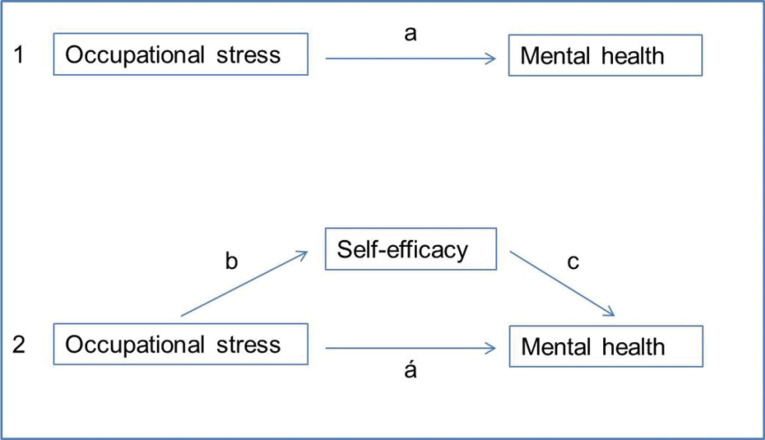
Graphic representation of the model testing the mediating role of self-efficacy on the association between occupational stress and mental health (MH). Note: 1 shows the theoretical association between occupational stress and MH, while 2 shows the theoretical model testing the mediating effect of self-efficacy on the association between occupational stress and MH

A mediation model was hypothesised to examine the effect of self-efficacy on the association between occupational stress and MH problems. In this model, occupational stress, MH problems, and self-efficacy acted as independent, dependent and mediator variables, respectively (Figure 1). Criteria for this mediation model were^23^: (1) the occupation stress should significantly predict the MH problems (path a), (2) the occupational stress should significantly predict the self-efficacy (path b), (3) the self-efficacy should significantly predict the MH problems (path c), and (4) the association between occupational stress and MH problems should be diminished (partial mediation) or no longer significant (full mediation) once self-efficacy is controlled (path á).

### Rationale

On the basis of the above considerations, study objectives were to examine: (1) the associations between occupational stress, MH problems, and self-efficacy among nursing professionals, and (2) the mediating effect of self-efficacy in the association between occupational stress and MH problems.

## Materials and Methods

### Study design and setting

The study design was cross-sectional and analytical. The study setting was 8 hospitals in Tabriz, Iran.

### Sample

Those nurses having an associate degree or higher in nursing, working for ≥1 year in their current job, with no history of mental disorders (by self-report) were considered to be eligible for the study. For structural equation modelling (SEM) studies, a sample size of at least 10 times greater than free parameters in the model has been recommended in the literature.^24^ Thus, at least 200 samples were determined to be required in this study to meet this criterion, considering 20 free parameters in the model. There were 1908 eligible nurses in the selected hospitals at the time of study. A two-stage sampling process was used. In the first stage, the hospitals were selected randomly and then in the next step, participants were selected according to the simple random sampling procedure. A total of 389 nursing staff participated in the study, which is much higher than the recommended sample size.

### Procedure

The authors made arrangements with participating hospitals for access permission. A questionnaire was administered for data collection including demographic and job characteristics (gender, age, education, marital status, job tenure, number of hours worked per day, work shifts, number of patients cared for, and taking on extra shifts) as well as occupational stress, self-efficacy and MH.

### Instruments and outcome measures

Occupational stress was assessed using the Health & Safety Executive (HSE) Management Standards Revised Indicator Tool (MS–RIT).^5^ This 35-item self-report questionnaire, with established reliability and validity,^3^ identifies psychosocial risks contributing to workplace stress. The advantages of the MS–RIT include its short length and ability to measure multiple dimensions of workplace stress.^5^ It has seven dimensions: demands (8 items assessing work procedures, work load, and work environment), control (6 items related to control and influence at work), managerial support (5 items assessing support and information provided by the employer), peer support (4 items related to support and encouragement from colleagues), relationships (4 items related to conflict and unacceptable behaviour at work), role (5 items regarding the job roles within the organisation) and change (3 items assessing organisational changes in the workplace). The items for demands and relationships dimensions are negatively phrased, which were reversed to help comparison across the other factors. Items responses are based on 5-point Likert-type scales (e.g., ‘always’ to ‘never’ and ‘strongly disagree’ to ‘strongly agree’), with higher scores showing lower levels of occupational stress. The Persian version of the MS–RIT, with good reliability and validity, was used in the study.^25^.

MH was evaluated by the general health questionnaire (GHQ–28), which is a well-tried and tested technique.^26^ This 28-item scale measures psychological (mental) distress in four areas: somatic symptoms, anxiety symptoms and sleep disorders, social dysfunction, and depression symptoms. Each subscale has 7 items and item scoring is based on a 4-point Likert scale (ranging from ‘never true’ = 0 to ‘always true’ = 3). Higher scores on this tool indicate more severe MH problems. A valid and reliable Persian version of this tool was used.^27^ MH problems was defined as GHQ–28 score ≥ 24.0.^27^

The 10-item general self-efficacy (GSE–10) scale was used to assess self-efficacy.^28^ This is a one-dimensional scale. Responses are based on a 4-point Likert scale (ranging from ‘totally incorrect’ to ‘totally correct’) (range = 10–40). Higher scores on this tool show higher levels of self-efficacy. A valid and reliable Persian version of the GSE–10 was used.^29^

### Data analysis 

First, correlations between all variables (continuous variable format) were estimated using Pearson’s correlation coefficients to determine the conditions necessary for assessing the mediation model.^23^ Then, the mediation model was examined using generalised structural equation modelling (GSEM) in Mplus v. 6.1.^30^ GSEM with WLSMV (weighted least squares mean and variance adjusted) was applied for this purpose. In this analysis, GHQ–28 score ≥ 24.0 and GHQ–28 <24 were considered for cases with and without MH problems, respectively. To achieve a model parsimony, those variables (e.g., demographic variables) that were not significantly contributed in the model, were eliminated. In the first step, the mentioned variables were tested and were removed from the model as they were not significant. In addition, a 2-step model building process proposed by Mulaik and Millsap^31^ was followed. However, as self-efficacy and MH are usually treated as the total score or their categories in the literature, and for discussion proposes, these variables were considered as observed variable, not latent variables. The following indices were used to assess the model fit: χ^2^/df < 5, root mean square error of approximation (RMSEA) <0.08, Tucker–Lewis index (TLI) >0.9, and comparative fit index (CFI) > 0.9. The significance level was considered as *P* < 0.05.

## Results

### Sample characteristics 

The majority of participants were females (81.7%), married (63.2%), and had undergraduate education (85.1%). Their mean (standard deviation – SD) age was 36.0 (6.9) years (range = 21–56 years), and the mean job tenure was 11.3 years (SD = 5.3 years). More details about job characteristics of the sample are presented in Table 1.

**Table 1:** Characteristics of the sample (n = 389)

**Variables**	
Gender, n (%)	
Male	71 (18.3)
Female	318 (81.7)
Age, years	
Mean (SD)	36.0 (6.9)
Range	21–56
Education, n (%)	
Undergraduate	331 (85.1)
Postgraduate	58 (14.9)
Marital status, n (%)	
Single	143 (36.8)
Married	246 (63.2)
Job tenure, years	
Mean (SD)	11.3 (5.4)
Range	1–30
Daily working time (h), n (%)	
≤8	211 (54.2)
>8	178 (45.8)
Work shifts, n (%)	
Morning	107 (27.5)
Evening	89 (22.9)
Night	135 (34.7)
Rotating	58 (14.9)
Number of patients cared for (individual nurse/shift), n (%)	
< 10	186 (47.8)
10–20	169 (43.4)
> 20	34 (8.8)
Taking on extra shifts, n (%)	
Yes	288 (74.0)
No	101 (26.0)

### Descriptive statistics 

The mean (SD) score of the MS–RIT was 109.2 (11.8) (range = 78–149). The demand subscale had the highest mean score (mean = 22.1; SD = 4.3), while the lowest mean score was related to change subscale (mean = 9.1; SD = 2.1). The Cronbach’s alphas were as follows: total MS–RIT (0.78), demand (0.81), control (0.69), managerial support (0.90), peer support (0.85), relationships (0.72), role (0.70), and change (0.74) (Table 2).

**Table 2:** Correlations among the variables

**Scale/Subscale**	**1)**	**2)**	**3)**	**4)**	**5)**	**6)**	**7)**	**8)**	**9)**	**10)**
1) Occupational stress (overall)	–									
2) Demand	0.543**	–								
3) Control	0.671**	0.216**	–							
4) Managerial support	0.750**	0.221**	0.489**	–						
5) Peer support	0.692**	0.225**	0.428**	0.579**	–					
6) Relationships	0.483**	0.401**	0.230**	0.229**	0.208**	–				
7) Role	0.419**	0.141**	0.205**	0.204**	0.284**	0.218**	–			
8) Change	0.747**	0.217**	0.517**	0.683**	0.537**	0.235**	0.284**	–		
9) Self-efficacy	0.356**	0.401**	0.408**	0.240**	0.217*	0.167*	0.306**	0.338**	–	
10) Mental health	-0.361**	-0.277**	-0.403**	-0.277**	-0.255**	-0.433**	-0.178*	-0.352**	-0.220**	–
Mean	109.2	22.1	18.1	15.1	13.2	11.3	20.1	9.1	17.7	22.5
(SD)	(13.4)	(4.3)	(3.6)	(3.9)	(2.5)	(2.8)	(2.9)	(2.1)	(4.9)	(10.2)

** *P* < 0.01 level (2-tailed).

The mean (SD) GHQ–28 score was 22.5 (10.2) (range = 3 – 61). A total of 163 (41.9%) participants had a GHQ–28 score higher than the cut-off point (e.g., those who scored ≥24.0 on GHQ–28). The Cronbach’s alpha was 0.81 for the GHQ–28.

The mean (SD) score of GSE–10 was 17.7 (4.9) (range = 10–40), indicating a relatively low level of self-efficacy among the studied nurses. The Cronbach’s alpha was 0.89 for the GSE–10.

### Correlation analysis

Significant correlations were found between the occupational stress, MH, and self-efficacy. The correlations were all significant and ranged from 0.140 to 0.684. Negative correlations existed between occupational stress and self-efficacy with MH, while the correlation between occupational stress and self-efficacy was positive (Table 2).

### GSEM modelling

**Figure 2:**
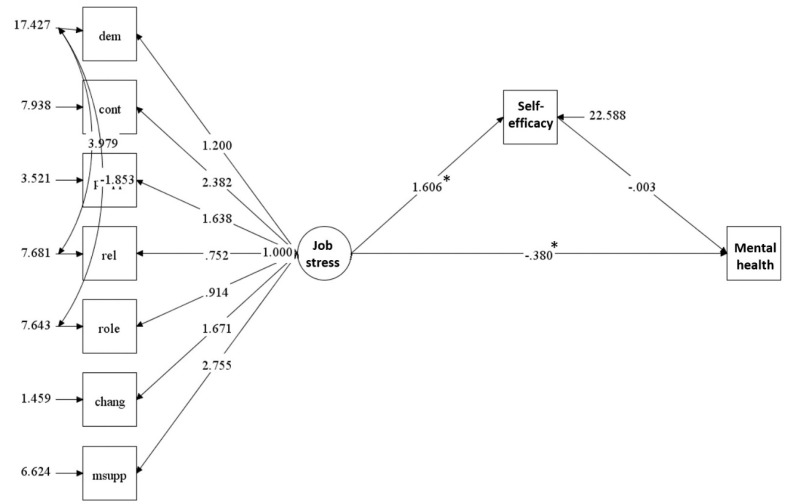
Conceptual model of the mediating role of self-efficacy on the association between occupational stress and mental health.
Note: DEM = Demand; CONT = Control; MSUPP = Managerial support; PSUPP = Peer support; RELAT = Relationships; ROLE = Role; CHANG = Change. Model fit indices: Chi-square (24, N=389) = 107.319, *P* < 0.001; normed chi-square (χ^2^/df) =4.34; RMSEA = 0.076; 90% CI = (0.068 to 0.104); CFI = 0.962; TLI = 0.958
*P < 0.001

A good fit to the data was obtained using the GSEM modelling: χ^2^ (24, N = 389) =104.319, *P* < 0.001; χ^2^/df = 4.34; RMSEA = 0.076; TLI = 0.962; CFI = 0.958. The results of structural modelling indicated that the path indicating direct effect of occupational stress on MH problems (path a) was significant (β = –0.38, *P* < 0.001). However, the path indicating indirect effect of occupational stress on MH problems through self-efficacy (e.g., the path between self-efficacy and MH or path b×c) was insignificant. These findings indicated that the self-efficacy did not mediate the association between occupational stress and MH problems (Figure 2).

## Discussion

The relationships among occupational stress, self-efficacy and MH problems, as well as the mediating effect of self-efficacy in the association between occupational stress and MH problems were investigated. The results revealed that occupational stress, poor MH, and low self-efficacy were fairly common among the study population, which might reflect the stressful and demanding nature of nursing job and underline the need for appropriate intervention strategies aimed at work condition improvements. The analyses also revealed that self-efficacy does not mediate the association between occupational stress and MH problems. These findings have possible implications with respect to employee health and patient outcomes.

In line with previous research, our findings demonstrate that job stress is a common problem among nursing personnel.^15,32-34^ However, it should be noted that the level of stress experienced by nurses in these studies varies greatly depending on the type of tools used for evaluation of stress, job type, and work setting. For instance, nurses working in intensive care units may experience higher levels of occupational stress than those studied in this research.^34^ This might be attributable to stressful working conditions in intensive care units such as high workload, unit space, and exposure to suffering patients or patient death.^35^ It is therefore crucial to consider the impact of variations in work load, work setting and work organisation to interpret the findings accurately. According to our findings, except for the role aspect, different dimensions of the MS–RIT contributed to experience of occupational stress among the studied nurses. This understanding of the experience of stress among nurses will help to develop appropriate preventive strategies to cope with this problem. In other words, workplace interventions aiming at improving job demands, control over the work situations, employee support (from both managerial and peer levels), relationships and changes are recommended with a view to helping prevent occupational stress in these employees.

It was shown that more than one-third of the studied nurses scored above the cut-off point for the GHQ, which is considerable. Some other studies have also reported a relatively high level of MH problems among nurses.^36-39^ These results provide further evidence that nurses are a group of healthcare professionals who are at risk for experiencing psychological problems. Again, this might reflect the nature of nursing job and emphasise the need for appropriate intervention strategies (with particular attention to the somatic symptoms, anxiety symptoms and sleep disorders as well as social dysfunctioning) to improve the MH status of these employees.

Our study hypothesis regarding significant association between occupational stress and MH problems was supported by the results. It was found that all domains of the MS–RIT were negatively related to MH status of nurses. This finding is not surprising in view of previous reports in this working population.^4,10,34,40^ The GSEM model result also supported an overall inverse association between occupational stress and self-efficacy in these employees. A very recent study also reported negative correlations between stress and self-efficacy among general nursing staff.^22^ It therefore appears that self-efficacy is an important factor influencing employees’ capability to deal with stressful conditions in the workplace.

One of the main contributions of this study is that the self-efficacy does not act as a mediator in the relationship between occupational stress and MH problems in nursing professionals. Some investigators have argued that employees with high level of self-efficacy have higher perception of control and that control is likely to moderate the association between stress and health.^41^ Therefore, it was plausible to hypothesise that the association between workplace stress and MH problems in nurses would be mediated by self-efficacy as an important individual trait. Nevertheless, this hypothesis was not supported by our results. In other words, despite the direct effect of occupational stress on MH problems, its indirect effect (e.g. through self-efficacy) was not statistically significant. Considering the multidimensional nature of occupational stress, this finding may imply that organisational and workplace stressors may have a more prominent role in the experience of MH problems in nurses than their individual traits (e.g., self-efficacy). However, it should be noted that workplace stressors vary greatly in different jobs and workplace settings. Therefore, further research is required to explore the generalizability of these findings to a variety of occupational settings and contexts. Nevertheless, the implication of this finding for nursing practice is that other factors than self-efficacy might be important in determining the association between workplace stress and MH problems in nursing personnel. Thus, additional research testing other mechanisms or variables that possibly mediate the association between occupational stress and MH problems are required to fill gap this area. To take account of this, consideration of other coping techniques (e.g., communication techniques, training, etc.) and other factors such as psychosocial (e.g., job satisfaction, burnout, rewards, leadership, etc.) and organisational (job organisational and design) factors, that have the potential to influence the association between workplace stress and MH, is recommended.

This is one of the first studies of its kind to investigate occupational stress, self-efficacy and MH problems and their relations among nursing staff. However, due to the transversal design of the study, there is a need for replication using prospective, longitudinal designs. It would also be necessary to replicate this study in other workplace settings or communities for generalisability purposes.

## Conclusion

The level of experienced stress, poor MH, and low self-efficacy in the studied nurses underscore the need for intervention programmes. The findings confirmed the hypothesis that occupational stress has direct effect on MH status of nurses. However, the indirect effect of stress through self-efficacy (e.g., mediating effect of self-efficacy) was not supported by our results. The findings highlight the role of other mechanisms or factors than self-efficacy in the association of workplace stress and MH that should be established in future work.

## Acknowledgments

The authors would like to acknowledge all subjects who participated in this study.

## Funding

No external funding sources were used for conducting the current research.

## Competing interests

Iman Dianat and Mohammad Asghari Jafarabadi are Associate Editors for Health Promotion Perspectives. Other authors declare that there is no conflict of interest.

## Ethical approval

This study was approved by the Tabriz University of Medical Sciences Ethics Committee (approval code: IR.TBZMED.REC.1395.825); all participating nurses signed a written informed consent form before participation.

## Authors’ contributions

ID contributed to the conception and work design as well as drafting the work. SA contributed to work design and data collection. FA contributed to the conception and work design. AB contributed to the data analysis and drafting the work. MAJ contributed to the analysis and interpretation of data.
